# QTOF and Orbitrap Technologies in Modern Pesticide Analysis: Applications in Residue Detection and Degradation Pathway Elucidation

**DOI:** 10.1155/jamc/7886371

**Published:** 2026-07-03

**Authors:** Ruilong Li, Liu Min, Kaili Gao, Liqun Zhong, Xingang Meng, Yue Cheng

**Affiliations:** ^1^ College of Materials Science and Engineering, Jingdezhen Ceramic University, Jingdezhen, Jiangxi, 333000, China, jci.edu.cn; ^2^ College of Biological and Environmental Engineering, Jingdezhen University, Jingdezhen, Jiangxi, 334000, China; ^3^ Guizhou Key Laboratory for Germplasm Innovation and Resource-Efficient Utilization of Dao-di Herbs, Bijie, 551700, China; ^4^ Bijie Institute of Traditional Chinese Medicine, Bijie, 551700, China

**Keywords:** degradation products, MS, Orbitrap, pesticide residue, QTOF

## Abstract

QTOF and Orbitrap techniques, as advanced mass spectrometry tools, show great potential in the field of pesticide residue analysis and degradation product identification. This paper reviews the application of these two techniques in pesticide residue analysis and degradation product identification. The principles and characteristics of QTOF and Orbitrap techniques are first introduced, including the advantages of high resolution, high sensitivity and high accuracy. Then, the applications of these techniques in different types of samples, including agricultural products, environmental samples and air samples, were explored. These studies demonstrate that QTOF and Orbitrap techniques can effectively detect and characterize low levels of pesticide residues and degradation products in complex samples. In addition, this paper summarizes the advantages and limitations of these techniques and suggests directions for future research. Taken together, the QTOF and Orbitrap techniques have important application prospects in pesticide residue analysis and degradation product identification and will provide strong support in areas such as food safety and environmental protection.

## 1. Introduction

The issue of pesticide residues has persistently posed a significant challenge to agricultural production and food safety. With the increase of pesticide use worldwide, there is an urgent need for the detection of pesticide residues and identification of degradation products in agricultural products [1]. In recent years, QTOF and Orbitrap technologies have shown great potential in this field.

First of all, conventional techniques for analysing pesticide residues are frequently hampered by laborious procedures, intricate sample preparation and a lack of sufficient sensitivity and selectivity. These methods are often unable to meet the requirements of accurate detection and quantification of multiple pesticide residues and cannot fully analyse the components in complex samples, and the emergence of QTOF and Orbitrap technologies has filled the gap, and their high resolution [2, 3], high sensitivity [4] and high accuracy [5] provide a new solution for pesticide residue analysis.

Second, the identification of pesticide degradation products [6] is important for assessing the environmental fate and toxicity of pesticides. However, traditional identification methods are often constrained by limited analytical scope and low sensitivity, making it difficult to accurately identify complex mixtures of degradation products [7], and the application of QTOF and Orbitrap technologies provides new possibilities [8–11] for the identification of pesticide degradation products, with its high resolution and high sensitivity, allowing a more comprehensive analysis of complex mixtures of degradation products and thus a more accurate assessment of the environmental risk of pesticides.

Although QTOF and Orbitrap technologies show great potential [12–15] for pesticide residue analysis and degradation product identification, they still face a number of challenges. These include the standardization of sample pretreatment, validation of analytical methods and optimization of instrument performance. However, it is believed that these challenges will be gradually overcome with the continuous development and improvement of the technologies, which will provide more reliable means and methods to solve the pesticide residue problems.

Therefore, this review will comprehensively introduce and analyse the latest research results of QTOF and Orbitrap technologies in pesticide residue analysis and degradation product identification, with a view to providing more effective technical support for the quality and safety of agricultural products and environmental protection.

## 2. Pesticide Monitoring

Pesticides are considered as substances or mixtures that are used to destroying pests and regulating plant growth in agriculture. They have been widely used in agricultural production areas in the past decades, substantially improving the productivity and quality of crops. At the same time, pesticides have been used in nonagricultural fields such as structural pest management, commercial vegetation control and companion animal care. However, the casual or heavy use of pesticides may lead to pest resistance to pesticides [16], human health problems [17] and environmental hazards [18].

Pesticides can be classified by chemical structure into four major categories: organochlorines (OCs), organophosphorus (OPs), carbamates and pyrethroids. OCs pesticides are highly toxic, and OCs are one of the most persistent pesticides, with a half‐life of about 10 to 30 years [19]. OPs pesticides and carbamates pesticides are affordable, short‐lasting and less toxic. But these two kinds of pesticides are cholinesterase inhibitors, which can damage the human central nervous system (CNS). Pyrethroid pesticides are synthetic pesticides modelled on the natural substance chrysanthemum esters [20]. However, the constant updating of pesticide species makes pesticide pollution dynamic, highly uncertain and complex.

Pesticide degradation is the only process that clears pesticides from the environment [21]. The degradation modes of pesticides include photolysis processes, chemical degradation processes such as hydrolysis and also biological degradation processes by microbial or plant action. Due to the abundance of pesticide species, there are a variety of possible degradation products of pesticides. Pesticide degradation products are often characterized by a wide variety and low concentration [22–24]. Consequently, the detection and identification of pesticide degradation products is more difficult.

In order to protect human health, pesticide concentrations in food and the environment need to be detected frequently. Due to the interference of complex matrices, low concentrations of degradation products and the lack of standard controls for degradation products, the analysis of pesticides and their degradation product identification is a complex, tedious and time‐consuming task. And this task still presents great technical challenges [25]. Currently, triple quadrupole mass spectrometry (Q‐MS) is the most widely employed method for detecting pesticides and their degradation products [26], Nevertheless, the types and quantities of pesticides that can be monitored are limited by the resolution and scanning speed of the instrument. With the development of technology, high‐resolution mass spectrometry (HRMS) [27] plays an increasingly important role in the determination of minor and trace pesticide degradation products in complex matrices such as the environment and food. At present, time‐of‐flight (TOF) [28] and Orbitrap mass spectrometry are commonly used for detecting pesticides and their degradation products, and they are usually used in tandem with quadrupoles to improve the accuracy of qualitative screening. Relatively speaking, Fourier transform ion cyclotron resonance (FTICR) [29] and magnetic sector mass spectrometry are less commonly employed for the analysis of pesticides.

## 3. Mass Spectrometry Techniques for Pesticide Analysis

### 3.1. Comparison of Common MS Techniques

Q‐MS [30] is widely used for pesticide detection in the environment and food because of its ease of operation and good quantitative ability. However, its accuracy is low in full scan mode, and the compounds are susceptible to matrix interference during ionization, which results in poor sensitivity and poor identification of compounds. In the selective ion detection mode, the sensitivity of the mass spectrometer has been improved, but it is still difficult to be used for the identification of compounds. Ion trap mass spectrometer (IT‐MS) [31] has multistage fragmentation (MSn) in full scan mode and higher sensitivity [32]. Therefore, unknown compounds can be identified by spectroscopy.

The triple quadrupole mass spectrometer (QQQ‐MS) [33–35] is extremely widely used for the quantitative detection of pesticides in complex matrices. The choice of different scanning modes (full scanning mode, parent ion scanning mode, selected ion scanning, neutral fragment loss scanning Neutral Loss, multireaction monitoring mode, etc.) gives the instrument a stronger quantitative ability and higher selectivity, and a certain qualitative ability, but it is sensitive to matrix interferences and has a low resolution, which still makes it difficult to identify the more complex unknown compounds. In the multireaction detection mode, the sensitivity and selectivity have been improved, but the contribution to the structural information of unknown compounds is still extremely limited [36]. And it is very difficult to identify unknown compounds from spectra in full scan mode due to poor sensitivity and other reasons.

HRMS mainly relies on precise mass numbers (which can be accurate to four decimal places) for qualitative identification. Compared with other types of mass spectrometry, it has the following three main advantages: (1) It possesses precise molecular weight determination, and the mass accuracy can be less than 2 ppm within a certain linear range so that matrix interference [37] can be excluded more thoroughly to realize higher selectivity. The elemental composition of parent and fragment ions can also be obtained, thus enabling the ability to identify unknown compounds and isotopes [38]. (2) Nontargeted qualitative screening and quantitative analysis can be achieved by the mass‐to‐charge ratio (m/z) information obtained in the full‐scan mode [39]. (3) Qualitative screening of pesticides and their degradation products by matching with HRMS databases reduces the dependence on pesticide controls and enables retrospective analysis of data [40]. In recent years, various HRMS techniques have been widely used in the identification of pesticide residues and their degradation products [41].

### 3.2. Advantages of QTOF and Orbitrap

The resolution of FTICR and magnetic mass spectrometry can reach 100 000, but due to their high cost, they are generally not used in the detection of pesticide residues. Currently, there is a greater tendency to use QTOF and Orbitrap two kinds of high‐resolution mass spectrometry.

**FIGURE 1:**
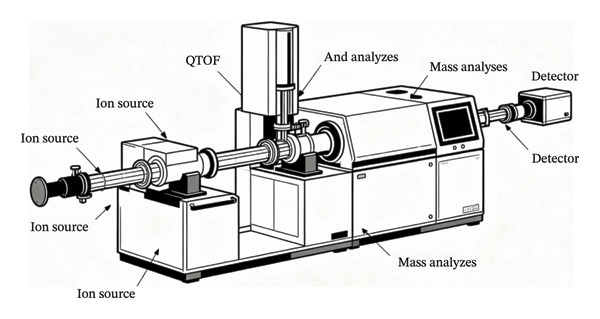
Schematic diagram of QTOF instrument.

Time‐of‐flight mass spectrometry (TOF‐MS) operates on the principle that ions possessing identical kinetic energy yet differing mass‐to‐charge ratios will separate by velocity as they traverse a field‐free flight tube, and the time it takes to travel a constant distance is different for the determination of the composition or structure of a substance. QTOF was first developed by Howard R Man et al. [42]. First developed in 1996, the hybrid quadrupole Q‐TOF instrument is composed of two quadrupole mass filters coupled with a time‐of‐flight (TOF) mass analyzer, and this configuration is widely applied in qualitative identification and precise mass determination of complex organic compounds. For the identification of pesticide degradation products, the first stage of Q‐MS selects a single ion and sends it to the second stage of quadrupole, where it undergoes collision‐induced dissociation (CID),and then arrives at the gas pedal of TOF‐MS, where it undergoes mass separation under the action of repulsor, thus realizing the precise molecular weight determination of the parent ions and fragments of ions [43]. QTOF has a resolution of 5000∼60,000 FWHM in general [44]. TOF‐MS has many advantages, such as fast scanning speed of microseconds [45], high ion transfer rate, high sensitivity, high resolution and theoretically no upper limit of the mass detection range [46], which makes it a mass spectrometer that plays a significant role in the identification of pesticide degradation products. However, the sensitivity of QTOF is easily affected by the resolution, resulting in a lower quantitative ability than QQQ [47]. Moreover, the instrument is susceptible to environmental influences. Schematic diagram of the QTOF instrument is shown in Figure 1.

**FIGURE 2:**
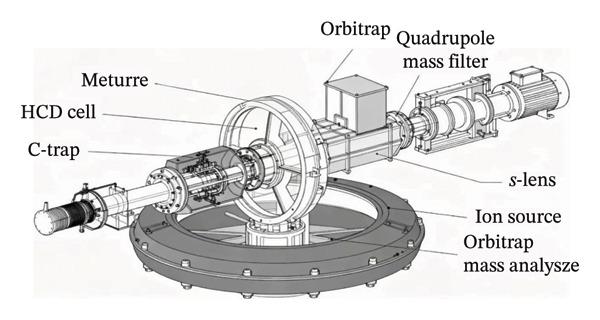
Schematic diagram of Orbitrap instrument.

Electrostatic field orbitrap mass spectrometry (Orbitrap) is a combination of electrostatic field ion trapping and fast Fourier transform techniques [48]. Orbitrap mass spectrometers have a mass analyser consisting of a spindle‐shaped central inner electrode and two outer spindle half electrodes on the left and right. By the action of the orbitrap trapping ions, the ions are made to rotate around the orbit of one centre electrode to reach a certain concentration in order to get enough sensitivity. The oscillation frequency of the ions is then determined by fast Fourier transform technique to calculate the mass‐to‐core ratio with a resolution up to 1,000,000 FWHM. Schematic diagram of the Orbitrap instrument is shown in Figure 2.

Compared with QTOF, EF‐Orbitrap mass spectrometry is a leader in resolution, mass accuracy, sensitivity, linear range and stability [49, 50]. Orbitrap sensitivity does not decrease with increasing resolution, and the instrument is generally unaffected by the environment. The orbitrap can be used in tandem with a quadrupole [51] to obtain both full‐scan mass spectra and resolution secondary mass spectra, or it can be used in tandem with a linear ion trap (LTQ) [52] for multistage mass spectrometry capability and more sensitive full‐scan detection.

### 3.3. Performance Comparison of QTOF and Orbitrap Technologies

There are differences between QTOF and Orbitrap technologies in terms of performance, such as resolution, mass accuracy, sensitivity and analysis speed. The resolution of QTOF technology is usually between 10,000 and 50,000, which can provide HRMS information with mass accuracy up to ppm level, which can meet the analysis needs of most pesticide residues and degradation products [53]. Its high analysis speed and the ability to complete multiple scans in a short period of time make it suitable for high‐throughput analysis. In terms of sensitivity, it has a good ability to detect common pesticide residues. However, when faced with complex matrix samples, the resolution may not be sufficient to accurately distinguish adjacent peaks.

Orbitrap technology has an ultrahigh resolution of more than 100,000 and a sub‐ppm mass accuracy, which makes it excellent for identifying subtle structural differences in pesticide degradation products. However, the speed of analysis is relatively slow, and it takes a long time to complete an analysis. At the same time, the cost of the instrument is higher, and the requirements for the experimental environment and operators are more stringent [54]. In general, QTOF technology is more suitable for high‐throughput routine pesticide residue analysis, while Orbitrap technology has advantages in scenarios that require high resolution and mass accuracy, such as accurate identification of pesticide degradation products.

In summary, QTOF and Orbitrap technologies have their own advantages in terms of performance, and their core advantages and disadvantages are summarized in Table 1.

**TABLE 1:** Comparison of QTOF and Orbitrap: advantages and limitations.

	QTOF	Orbitrap
Resolution	Moderate (typically 10,000–60,000 FWHM)	Ultrahigh (up to > 100,000 FWHM, and even 1,000,000)
Quality accuracy	High (up to ppm level)	Extremely high (up to sub‐ppm level)
Speed of analysis	Very fast and suitable for high‐throughput screening	Relatively slow
Sensitivity	High, but may decrease with increasing resolution	High, and the sensitivity does not decrease with increasing resolution
Quantitative capabilities	Good, but generally not as effective as the triple quadrupole	Good, wide linear range
Main advantages	Fast scanning speed, high throughput analysis capability, suitable for unknown screening and advantages for volatile and semivolatile compound analysis	It has high resolution and mass accuracy, good stability and is suitable for accurate identification and discrimination of structural similarities in trace substances in complex matrices
Main limitations	The resolution is relatively low, and adjacent mass peaks may not be resolved in complex matrices	Slow analysis speed; higher instrument acquisition and O&M costs; more demanding requirements for operators and experimental environments
Applicable scenarios	High‐throughput screening of conventional pesticides with multiple residues and nontargeted screening of unknowns	Ultrahigh resolution confirmatory analysis, accurate identification of degradation products/conversion products and sample analysis with high interference with complex matrices are required

### 3.4. Combined QTOF‐Orbitrap Applications

The joint application of QTOF and Orbitrap technology has strong feasibility. From the principle level, QTOF technology can quickly obtain HRMS information and achieve high‐throughput analysis based on the mass screening of quadrupoles and accurate TOF measurement. Orbitrap technology uses the movement of ions in the orbitrap for mass analysis with ultrahigh resolution and mass accuracy. There is no conflict between the two principles, and the samples can be analysed from different angles, which can form a complementary data.

In terms of instrument compatibility, modern mass spectrometry instruments are increasingly designed with a focus on openness and compatibility, and QTOF and Orbitrap mass spectrometers can be connected to the same front‐end separation equipment (e.g., liquid chromatograph and gas chromatograph) for easy integration into the same analytical system [55]. In addition, the supporting software system can also manage and analyse the data generated by the two technologies in a unified manner, providing technical support for joint applications.

The combined application has significant advantages in the analysis of pesticide residues and the identification of degradation products. It can improve the accuracy and reliability of the analysis. QTOF technology excels in high‐throughput analysis, allowing for rapid primary screening of large quantities of pesticide residues and providing abundant mass spectrometry information. The ultrahigh resolution and mass accuracy of Orbitrap technology can further confirm and accurately quantify the results obtained from the preliminary screening of QTOF and can more accurately distinguish and identify pesticides and their degradation products with similar structures, effectively reducing the probability of false positive and false negative results [56, 57]. Different pesticides and degradation products have different physicochemical properties, and there may be blind spots in detection of a single technology. QTOF technology has advantages for the detection of some volatile and semivolatile pesticides, while Orbitrap technology performs better in the analysis of pesticides and their degradation products with strong polarity and poor thermal stability. The combination of the two can cover a wider range of pesticide types and degradation products, improving the comprehensiveness of detection.

In the actual analysis of pesticide residues, the sample matrix is often very complex and can interfere with the analysis results. The combination of QTOF and Orbitrap technologies, combined with advanced sample preparation methods and data analysis algorithms, can more effectively remove matrix interferences, improve detection sensitivity and accuracy and better cope with the analysis needs of complex samples.

## 4. QTOF and Orbitrap Applications in Pesticide Analysis

### 4.1. Chromatographic Coupling Methods

QTOF and Orbitrap technologies are characterized by high sensitivity, high resolution and high accuracy, which can effectively analyse pesticide residues in food and can identify and confirm various residues in complex samples. The application of these two technologies in pesticide residue detection provides powerful technical support to improve the accuracy and efficiency of detection. Their HRMS data can provide detailed analytical information, including molecular formulae and fragmentation profiles, thus realizing accurate identification and quantitative analysis of the residues.

The combination of QTOF and GC or LC is widely used and important in pesticide residue detection [58–60]. By combining QTOF with GC or LC, the advantages of both can be fully utilized to improve the efficiency and accuracy of pesticide residue analysis.

First of all, GC and LC are commonly used techniques for pesticide residue analysis, and GC is mainly applicable to the analysis of pesticide residues with high volatility and high thermal stability, while LC is mainly applicable to the analysis of pesticide residues with poor polarity and thermal stability. By coupling with QTOF, GC or LC can effectively separate and purify the pesticide residues in the sample and direct them to QTOF for HRMS analysis. Second, the main advantage of GC or LC coupled with QTOF is that it provides more accurate, sensitive and reliable results for pesticide residue analysis. QTOF is characterized by high resolution, high sensitivity, wide linear range and accurate mass determination and is capable of providing detailed mass spectrometry data including molecular formulae, fragmentation profiles and retention times. Compared with traditional mass spectrometers, QTOF can provide more comprehensive and detailed pesticide residue analysis results and can accurately identify and quantify multiple pesticide residues in complex samples.

### 4.2. Application in Different Matrices

The importance of pesticide residue testing in food cannot be overstated, as it is directly related to public health and food safety. Pesticides are chemical substances commonly used in agricultural production to protect crops from pests and diseases, but excessive or improper use of pesticides may result in their residues in food, posing a potential threat to human health. Therefore, timely and accurate detection and monitoring of pesticide residue levels in food has become one of the most important measures to protect food quality and public health [61].

Kottadiyil, Divya et al. [62] used GC‐MS/MS and UHPLC‐QTOF MS for the detection of 52 pesticide residues in vegetables and fruits on a matrix consisting mainly of tomatoes, eggplants, bananas, pomegranates and oranges. The QuEChERS technique and d‐SPE method were used for pretreatment. Dietary risk assessment was also performed on various food products. The results showed that the overall is safe, but there are a small number of pesticide residues. Pedersen et al. [63] developed a rapid detection of pesticide residues in pig muscle by LC‐QTOF, the pretreatment was by solid‐phase extraction, and the detection was by HRMS. The stability of pesticide residues under different storage conditions was investigated. Pang Xu et al. [64] developed a gas chromatography with quadrupole orbitrap mass spectrometry (GC‐Orbitrap‐MS) method for the detection of 191 pesticide residues in beef with a modified QuEChERS pretreatment. The results showed that the method has excellent sensitivity, linearity, accuracy and precision. Qu, L et al. [65] established a LC‐Q Orbitrap method for the determination of pesticide residues in eggs. The pretreatment was QuEChERS method with on‐line solid phase extraction. The method validation results showed that the method has a low detection limit and high precision. Bang Han Yeol et al. [66] developed a simultaneous multicomponent analysis of 12 high‐frequency detection pesticides in bell pepper based on ultraperformance liquid chromatography quadrupole time‐of‐flight mass spectrometry (UHPLC QTOF) combined with QuEChERS pretreatment and validated the method. The results showed that QuEChERS preparation simplified sample preparation and reduced the amount of organic solvent used, while maintaining high recovery and low interference.

**FIGURE 3:**
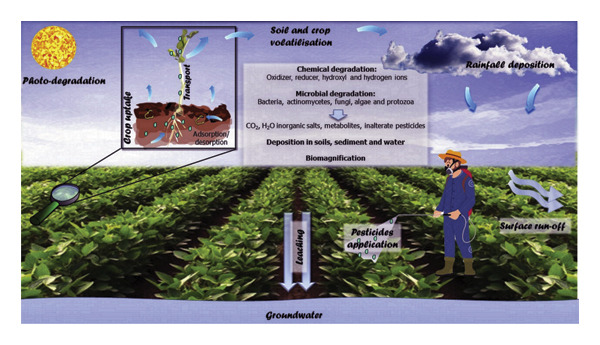
Environmental fate of pesticides in water matrices and corresponding monitoring strategies, illustrating the pathways from agricultural application to environmental detection and risk assessment. Source: adapted from Campanale et al. [67].

Pesticide residues in the environment may lead to a variety of problems, including soil and water contamination, damage to nontarget species, ecosystem destruction and accumulation in the food chain. Therefore, timely detection and monitoring of pesticide residues in the environment can help to take effective measures to reduce their negative impacts on the environment. Figure 3 provides a comprehensive overview of pesticide pathways in aquatic environments and the corresponding monitoring strategies, highlighting the critical role of advanced analytical techniques in understanding environmental fate and implementing effective remediation measures. QTOF and Orbitrap technologies, as high‐resolution and high‐sensitivity mass spectrometry analytical tools, are effective in detecting and characterizing pesticide residues and degradation products at trace levels in the environment. Their advantages include high accuracy, reliability and the ability to detect multiple pesticides and degradation products simultaneously. Therefore, these advanced techniques have important application prospects in the field of environmental monitoring and food safety, which can help to protect the environment and maintain the ecological balance, as well as safeguard human health.

**FIGURE 4:**
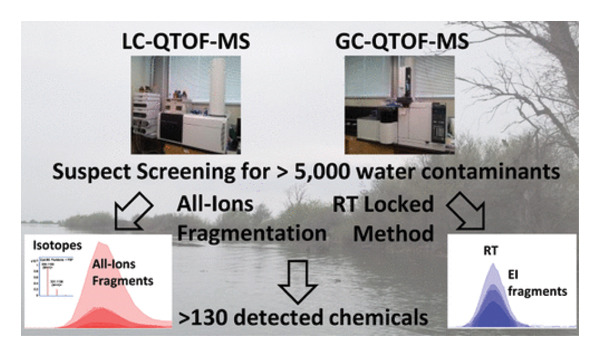
Representative chromatographic profiles obtained from the analysis of environmental water samples using LC‐/GC‐QTOF‐MS, showing the complementary detection of a wide range of micropollutants. Source: adapted from Moschet et al. [68].

Moschet Christoph et al. [68] applied both LC‐QTOF‐MS and GC‐QTOF‐MS for the screening of a wide range of compounds in the Sacramento‐San Joaquin River Delta, respectively. The results showed that both coupling methods screened the target substances well, and the combination of liquid chromatography and QTOF has a wider range of applications. Figure 4, adapted from Moschet et al. (2017), displays chromatographic profiles from a water sample analysis, clearly demonstrating the capability of QTOF‐MS to detect and identify trace‐level micropollutants in a complex environmental matrix. Mun Ju Jeong et al. [69] developed an analytical method for the simultaneous detection of multiple residues of 504 pesticides in a variety of crops using UHPLC QTOF and integrated MS1 and MS2 levels through sequential window acquisition of all theoretical mass spectrometry levels to enhance pesticide residue identification. Gómez‐Ramos et al. [70] demonstrated that analytical methods based on GC‐TOF‐MS and GC‐Orbitrap‐MS are adaptable and effective in screening nontargeted environmental pollutants. And the data can be recorded in a database so that the follow‐up work can be alleviated accordingly. Casado J. D et al. [71] established an LC‐Q‐Orbitrap‐MS method for the detection of 252 pesticide residues in surface water, and the pretreatment was based on solid phase extraction. Yuanyuan Wang et al. [72] analysed 352 pesticide residues in chrysanthemums by GC‐Orbitrap‐MS and used a simplified pretreatment method of one‐step extraction and dilution to perform qualitative and quantitative detection in the full‐scan mode. The results showed that the method had a low detection limit and good quantitative and qualitative abilities.

However, there are some drawbacks and challenges in the coupling of QTOF with GC or LC. First, the analytical method of the coupling is complicated, requiring specialized operators and longer analysis time. Second, the cost of instrumentation and consumables for the coupling is high, which brings some difficulties to the operation and maintenance of the laboratory. In addition, the coupling method requires high requirements for sample pretreatment and sample preparation, and it is necessary to choose appropriate extraction, purification and derivatization methods to obtain reliable analytical results. Currently, QTOF coupled with GC or LC is widely used in the field of pesticide residue detection. According to the characteristics and analytical requirements of different pesticide residues, suitable GC or LC methods can be selected for sample separation and purification and then QTOF can be used for HRMS analysis. This coupled method has made significant progress in the accuracy and sensitivity of pesticide residue analysis and provides powerful technical support for improving food safety and public health.

## 5. Identification of Pesticide Degradation Products

### 5.1. Environmental Pesticide Degradation Products

In the atmosphere, pesticides may be affected by factors such as light, oxidation and photolysis, leading to the production of various degradation products. Degradation in water may be influenced by water chemistry, microbial activity and environmental pH. Degradation in soil may be influenced by factors such as soil type, moisture, temperature and microbial activity.

These degradation characteristics present a series of detection difficulties. The first is the complex sample matrix, the atmosphere, water and soil samples may have a variety of organic and inorganic components: these matrices may interfere with the pesticide degradation products, making the detection and identification of degradation products complicated. Second is the low concentration of the target: in the degradation process, the pesticide may be gradually degraded into a lower concentration of products; these products may exist in very low concentrations in the sample, so the need for high sensitivity detection methods. Finally, the structural complexity: pesticide degradation products may have many different structures, involving a variety of possible degradation pathways and reaction products, thus requiring analytical methods capable of accurate identification and structural resolution.

QTOF and Orbitrap technologies provide an effective means of overcoming these detection difficulties through their high resolution, high sensitivity and ability to perform accurate mass determination. These techniques provide a wealth of mass spectral information, including the precise mass of the molecular ions, the mass‐to‐charge ratio of the fragment ions and the relative abundance relationships between the fragment ions, allowing for the accurate identification and structural resolution of degradation products in samples. In the atmosphere, pesticides may be affected by factors such as light, oxidation and photolysis, leading to the production of various degradation products. Degradation in water may be influenced by water chemistry, microbial activity and environmental pH. Degradation in soil may be influenced by factors such as soil type, moisture, temperature and microbial activity.

### 5.2. Application in Different Matrices

The identification of pesticide degradation products is equally important. Pesticides undergo degradation processes after exposure or use in the environment, resulting in a variety of degradation products. These degradation products may have different toxicities, biological activities and environmental behaviours and may have potential impacts on ecosystems and human health.

Therefore, the accurate characterization of pesticide degradation products is essential for a comprehensive understanding of the fate and effects of pesticides in the environment. Complex pesticide degradation products can be efficiently identified and quantitatively analysed through the use of HRMS techniques such as QTOF and Orbitrap. The high sensitivity and resolution of these advanced techniques allow for the accurate analysis and identification of degradation products at trace levels in complex environmental matrices.

The identification of pesticide degradation products allows for a better assessment of pesticide stability, toxicity and bioavailability in the environment, thereby guiding rational pesticide use and environmental protection measures. In addition, knowledge of pesticide degradation products can help to develop effective environmental monitoring and management strategies to protect the stability of ecosystems and minimize their potential risks to the environment and human health. Therefore, the identification of pesticide degradation products is emphasized in the review as essential for a comprehensive understanding of the behaviour and effects of pesticides in the environment.

**FIGURE 5:**
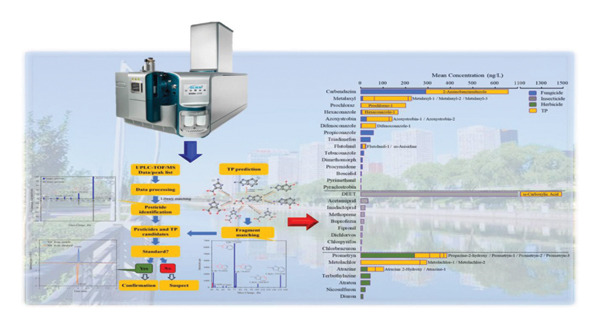
Representative extracted ion chromatograms (XICs) obtained by LC‐QTOF‐MS analysis of a river water sample, showing the detection of selected pesticides and their transformation products. Source: adapted from Zhang et al. (2021).

Pang Guofang et al. [73] in 2020 used GC/LC‐Q‐TOFMS combination technique to simultaneously screen 733 pesticides in fruits and vegetables on substrates including bell peppers, carrots, cauliflower, leeks, mangosteen and durum, and the pretreatment was mainly based on solid phase extraction. An accurate quality database was also applied for comparison during the screening process. The results showed that this technique has a unique advantage of integrating the advantages of the two kinds, with high accuracy and precision of the detection results. Cha Kyung Hoon et al. [74] established a GC‐APCI Q‐TOF method for quantitative screening of pesticides and designed a new APCI dopant system. It was used for pesticide identification of standard solutions containing 415 pesticides, and 320–340 pesticides were finally identified from the standard solutions. The results showed that the technique has good ability to identify pesticide residues. Makni Yassine et al. [75] developed a screening method based on QuEChERS and UHPLC‐Q‐TOF for the multiresidue analysis of pesticides in baby food. They utilized specific thresholds for retention time and exact mass accuracy to minimize false positives and negatives and further confirmed results by comparing against known fragmentation spectra. The results showed a screening detection line of 95% and a limit of identification of 73%, which provides good pesticide identification, i.e., the ability to screen for unknown pesticides. Drakopoulou, Sofia et al. [76] developed an LC‐(ESI)‐/GC‐(APCI)‐QTOF MS method for the detection of 771 pesticides in olive oil. The method was validated in terms of precision, accuracy and matrix effect by comparing the two coupling methods separately. The results showed a wider range of identification with LC coupling. Zhang, Yang et al. [77] used LC‐QTOF‐MS to detect and screen pesticide residues and degradation products in rivers. A database of 557 commercial pesticides and more than 1400 predicted transformation products (TPs) was used for nontargeted screening. Twenty TPs and 30 pesticides were finally identified. As shown in Figure 5, the extracted ion chromatograms clearly demonstrate the detection of several key pesticides and their TPs in a complex river water sample, highlighting the powerful separation and identification capability of the LC‐QTOF‐MS system. The results showed that the method can be well used for the identification of suspicious compounds with good identification results. Jian Wang et al. [78] used ultraperformance liquid chromatography electrospray Q Orbitrap mass spectrometry (UHPLC/ESI Q Orbitrap) combined with the QuEChERS method to quantify 655 pesticide residues in fruits and vegetables and evaluated two methods for data sorting and identification of isomers and isobaric ions. It provides a highly sensitive and specific solution for multipesticide residue screening, especially for isomer and metabolite analysis.

Wang, Jian et al. [79] developed an analytical method for the determination of 451 pesticide residues in fruits and vegetables by UHPLC/ESI Q‐Orbitrap MS. The pretreatment was performed by the QuEChERS method, and the standard curve was established using the internal standard method. The proximity side and screening ability of the method was verified by overall recovery, accuracy and precision. UHPLC/ESI Q‐Orbitrap showed great potential for identification in experiments and is being further investigated in routine practice. Kosma, Christina I. et al. [80] developed an analytical approach using QuEChERS and UHPLC‐Orbitrap‐MS for the detection of pesticides and their degradation products in wine. Initial identification relied on accurate mass and retention time, followed by confirmation through MS fragmentation. The method’s performance was rigorously validated by determining its LOD, LOQ, linearity, recovery, precision and matrix effect. Finally, the validated method was successfully deployed on real wine samples. Mol Hans GJ et al. [81] evaluated the capability of GC‐EI Orbitrap HRMS for pesticide residue analysis and degradation product identification, using pesticides in fruits and vegetables as a model. Following a QuEChERS pretreatment, the method was validated for 54 pesticides across three matrices (tomato, leek and orange) and verified according to the EU GC‐HRMS standard (SANTE/11945/2015). The results demonstrated that the GC‐EI full‐scan Orbitrap HRMS is highly suitable for quantitative residue analysis and possesses excellent qualitative screening capabilities. Feng Chao et al. [82] analysed pesticide multiresidues in food samples using GC‐MS/MS and UPLC‐Q‐Orbitrap. The combination of full scan and data independent acquisition modes was evaluated in real samples to improve and facilitate the pesticide screening process, and the results showed that various pesticide residues were detected with high detection rates (> 90%) and satisfactory recoveries (70%–130%), and no false positive results were reported. Hakme Elena et al. [83] used a cleaned‐up microsolid‐phase extraction system in conjunction with a robotic autosampler combined with GC‐Orbitrap‐MS to analyse pesticide residues in cereals. The spike recoveries of all pesticides were in the range of 70%–120%, and the reproducibility (calculated as the relative standard deviation) was less than 20%. The results showed that the method has good quantitative and qualitative abilities.

M. Riva et al. [84] were the first to make a side‐by‐side comparison of chemical ionization Orbitrap mass spectrometry (CI Orbitrap) and the widely used atmospheric pressure interface time‐of‐flight mass spectrometry (CI APi TOF), using two different chemical ionization methods, to explore the ability of CI Orbitrap to accurately measure the low concentrations of gaseous species formed by the oxidation of *α* pinene and to achieve accurate quantification of organic compounds as low as 1 × 10^5^ molecules cm^−3^ through newly developed linear correction. This method can be extended to other bioderived volatile organic compounds (BVOCs) oxidation studies, providing molecular‐level data support for aerosol nucleation and growth mechanisms.

## 6. Conclusions

In this review, we comprehensively discuss the application of QTOF and Orbitrap mass spectrometry techniques in pesticide residue analysis and degradation product identification. These advanced methods offer high sensitivity, resolution and reliability, enabling effective detection and characterization of trace levels of pesticides and their TPs across diverse sample types—including agricultural, environmental and air samples—while supporting multiresidue analysis. Despite their robust performance across matrices, challenges remain in method standardization, matrix effect management and data interpretation. Future efforts should address these issues to further expand the role of these techniques in supporting pesticide regulation, environmental protection and food safety.

## Author Contributions

Ruilong Li: writing–original draft, methodology and investigation. Liu Min: validation, supervision and data curation. Kaili Gao: validation. Liqun Zhong: investigation, software and formal analysis. Xingang Meng and Yue Cheng: writing–review and editing, supervision and funding acquisition.

## Funding

This study was funded by the Science and Technology Research Project of the Education Department of Jiangxi Province, GJJ2402301; the Science and Technology Research Project of the Education Department of Jiangxi Province, GJJ2202411; Subject Construction Project of Jingdezhen Science and Technology Plan, 20202GYZD015‐07; and the National Innovation and Entrepreneurship Training Program for College Students of Jingdezhen University, 202410894009.

## Conflicts of Interest

The authors declare no conflicts of interest.

## Data Availability

Data supporting the findings of this study are available from the corresponding authors upon reasonable request.
